# Evaluation of myCOPD Digital Self-management Technology in a Remote and Rural Population: Real-world Feasibility Study

**DOI:** 10.2196/30782

**Published:** 2022-02-07

**Authors:** Rowena Cooper, Adam Giangreco, Michelle Duffy, Elaine Finlayson, Shellie Hamilton, Mahri Swanson, Judith Colligan, Joanna Gilliatt, Mairi McIvor, Elizabeth Kathryn Sage

**Affiliations:** 1 Department of Respiratory Medicine National Health Service Highland Inverness United Kingdom; 2 Division of Biomedical Sciences University of the Highlands and Islands Inverness United Kingdom; 3 Specialist Community Respiratory Nursing Team National Health Service Highland Inverness United Kingdom; 4 Technology Enabled Care Team National Health Service Highland Inverness United Kingdom; 5 Institute for Applied Health Sciences Centre for Rural Health University of Aberdeen Inverness United Kingdom

**Keywords:** digital self-management, COPD, remote and rural, mobile health, application, chronic pulmonary obstructive disease, rural communities

## Abstract

**Background:**

Chronic obstructive pulmonary disease (COPD) is a common, costly, and incurable respiratory disease affecting 1.2 million people in the United Kingdom alone. Acute COPD exacerbations requiring hospitalization place significant demands on health services, and the incidence of COPD in poor, remote, and rural populations is up to twice that of cities.

**Objective:**

myCOPD is a commercial, digital health, self-management technology designed to improve COPD outcomes and mitigate demands on health services. In this pragmatic real-world feasibility study, we aimed to evaluate myCOPD use and its clinical effectiveness at reducing hospitalizations, inpatient bed days, and other National Health Service (NHS) resource use.

**Methods:**

myCOPD engagement and NHS resource use was monitored for up to 1 year after myCOPD activation and was compared against health service use in the year prior to activation. A total of 113 participants from predominantly remote and rural communities were recruited via community-based care settings, including scheduled home visits, outpatient appointments, pulmonary rehabilitation, and phone or group appointments. There were no predetermined age, disease severity, geographical, or socioeconomic inclusion or exclusion criteria.

**Results:**

Out of 113 participants, 89 activated myCOPD (78.8%), with 56% (50/89) of those participants doing so on the day of enrollment and 90% (80/89) doing so within 1 month. There was no correlation between participant enrollment, activation, or myCOPD engagement and either age, socioeconomics, rurality, or COPD severity. Most active participants used at least one myCOPD module and entered their symptom scores at least once (79/89, 89%). A subgroup (15/89, 17%) recorded their symptom scores very frequently (>1 time every 5 days), 14 of whom (93%) also used four or five myCOPD modules. Overall, there were no differences in hospital admissions, inpatient bed days, or other health service use before or after myCOPD activation, apart from a modest increase in home visits. Subgroup analysis did, however, identify a trend toward reduced inpatient bed days and hospital admissions for those participants with very high myCOPD usage.

**Conclusions:**

Our results suggest that neither age, wealth, nor geographical location represent significant barriers to using myCOPD. This finding may help mitigate perceived risks of increased health inequalities associated with the use of digital health technologies as part of routine care provision. Despite high levels of activation, myCOPD did not reduce overall demands on health services, such as hospital admissions or inpatient bed days. Subgroup analysis did, however, suggest that very high myCOPD usage was associated with a moderate reduction in NHS resource use. Thus, although our study does not support implementation of myCOPD to reduce health service demands on a population-wide basis, our results do indicate that highly engaged patients may derive benefits.

## Introduction

Chronic obstructive pulmonary disease (COPD) is a common, costly, and incurable respiratory disease affecting 1.2 million people in the United Kingdom alone. Annually, it costs the National Health Service (NHS) £1.9 billion (US $2.43 billion), it requires over 1 million inpatient bed days due to acute exacerbations requiring hospitalization, and it places significant demands on health services [1-3]. The prevalence of COPD in poor, remote, and rural populations is twice that of cities [4].

Effective COPD self-management can reduce both exacerbation-induced hospital admissions and health service use when compared to standard care [5,6]. myCOPD is a digital health self-management technology designed to improve COPD outcomes and mitigate demands on services [7]. myCOPD modules include symptom scoring, inhaler technique, and a virtual pulmonary rehabilitation course. Previous studies indicate that myCOPD is associated with reduced inhaler technique errors, lower COPD Assessment Test (CAT) scores, and fewer hospital readmissions within 3 months of an exacerbation [8-11].

NHS Highland covers the largest geographical area of Scotland, contains regions of significant socioeconomic deprivation, and has a majority remote and rural population. Access to hospital services and delivering equity of care remains challenging, and digital health technologies represent one potential solution. In this pragmatic test-of-change study, we evaluated myCOPD and its effectiveness at reducing hospitalizations, inpatient bed days, and other health service use.

## Methods

### Study Design

This was a 1-year, longitudinal, test-of-change evaluation of the digital self-management technology myCOPD for patients with COPD. The study received Caldicott Guardian approval for anonymized health record data analysis; it received internal ethical approval by NHS Highland Research, Development & Innovation; and all participants provided written informed consent.

### Participants

Participants were recruited over a 6-month period, from May to October 2019, as part of routine community-based care, including scheduled home visits, outpatient appointments, pulmonary rehabilitation, and phone or group appointments. As this was a pragmatic real-world assessment of myCOPD, there were no predetermined age, disease severity, geographical, or socioeconomic inclusion or exclusion criteria. Participants lacking appropriate digital devices, technological skills, or connectivity were not enrolled. A total of 140 people throughout the Scottish Highlands with COPD were offered myCOPD, of whom 120 enrolled during routine health care encounters, including scheduled home visits (n=54, 45.0%), outpatient appointments (n=43, 35.8%), pulmonary rehabilitation (n=13, 10.8%), and phone or group appointments (n=10, 8.3%).

### Intervention

Once enrolled, participants activated the technology by registering and creating an account on the myCOPD platform, which was accessed via an email link sent to each participant. Up to four reminders were sent on a weekly basis to encourage myCOPD activation. Participants used the technology as they wished and did not receive further encouragement during the evaluation. Participants were provided with licenses at no cost to themselves.

### Data Analysis

All participant data were collected for the 12-month period prior to myCOPD enrollment and up to 12 months following technology activation. myCOPD engagement data were collected via the myCOPD clinician portal. Health service use data were obtained via NHS electronic care records, including the NHS Highland Clinical Portal system and out-of-hours contacts using Adastra. Participant rurality and Scottish Index of Multiple Deprivation (SIMD) data were calculated using participant postcodes and relevant lookup tables [12,13].

Health service data were evaluated on a longitudinal basis for all enrolled participants, comparing the incidence of daily hospital admissions, inpatient bed days, and other service use for the period before and after myCOPD activation to March 1, 2020. Enrollment was defined as a participant who consented to participate, received an invitation to enroll, and was allocated a myCOPD license. Activation was defined as a participant who accessed the myCOPD platform and completed account registration. Symptom scoring frequency was defined as follows: low (<1 time every 100 days), moderate (1-5 times every 100 days), high (6-20 times every 100 days), and very high (>20 times every 100 days). Health care usage data from participants who enrolled but did not activate myCOPD contributed to “before” activation results. All data were compliant with the General Data Protection Regulation.

### Statistical Analysis

Power calculations determined that a study size of 100 participants was sufficient to evaluate a primary endpoint of reduced inpatient bed days. Calculations were based on projected modest (10%) reductions in inpatient bed days and significant (25%) seasonal variability in COPD exacerbations, with significance (α) at .05 and 80% power (1-β). Paired participant health care usage data before and after myCOPD activation were analyzed using the Wilcoxon signed-rank test. Statistical analysis was not performed on user subgroups representing variable myCOPD symptom scoring frequency or module usage, as subgroups were not sufficiently powered.

## Results

**Figure 1:**
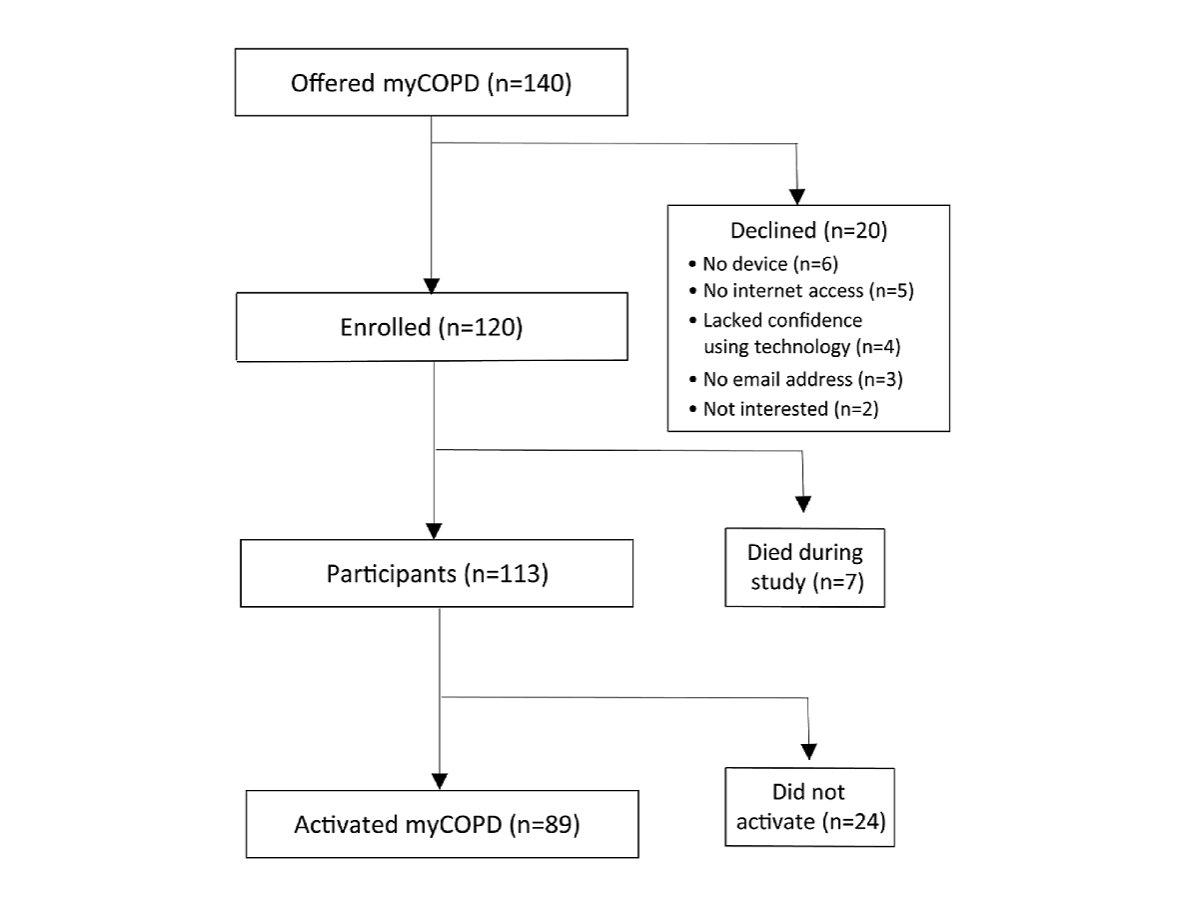
CONSORT (Consolidated Standards of Reporting Trials) flowchart of study participants who were offered myCOPD, showing the number of patients who declined and reasons why, the number enrolled, and the number included in final study. COPD: chronic obstructive pulmonary disease.

Of the 140 people invited to enroll in myCOPD, 20 (14.3%) declined to participate, mostly for technology-related reasons. Of the remaining 120, 7 (5.8%) were excluded, as they died during the study period, leaving a total of 113 participants (Figure 1). The average participant age was 69.3 (SD 8.2) years, and 51.3% (58/113) were female. A total of 70.8% (80/113) of participants were from remote and rural areas, and 75.2% (85/113) represented the three most deprived SIMD quintiles. Most participants (69/113, 61.1%) had moderate or severe COPD, and 20.4% (23/113) had very severe disease according to their Global Initiative for Chronic Obstructive Lung Disease score (Table 1) [14].

**Table 1:** myCOPD^a^ participant characteristics.

Participant characteristics			Participants (N=113), n (%)
**Age in years**			
	31-40	0 (0)	
	41-50	2 (1.8)	
	51-60	21 (18.6)	
	61-70	37 (32.7)	
	71-80	35 (31.0)	
	≥81	5 (4.4)	
	Not recorded	13 (11.5)	
**Sex**			
	Female	58 (51.3)	
	Male	55 (48.7)	
**Socioeconomics (SIMD^b^ quintile)**			
	1 (most deprived)	11 (9.7)	
	2	27 (23.9)	
	3	47 (41.6)	
	4	25 (22.1)	
	5 (least deprived)	3 (2.7)	
**Urban-rural classification**			
	Large urban areas	0 (0)	
	Other urban areas	31 (27.4)	
	Accessible small towns	2 (1.8)	
	Remote small towns	26 (23.0)	
	Accessible rural areas	7 (6.2)	
	Remote rural areas	47 (41.6)	
**COPD severity**			
	Mild	11 (9.7)	
	Moderate	33 (29.2)	
	Severe	36 (31.9)	
	Very severe	23 (20.4)	
	Not recorded	10 (8.8)	

^a^COPD: chronic obstructive pulmonary disease.

^b^SIMD: Scottish Index of Multiple Deprivation.

**Figure 2:**
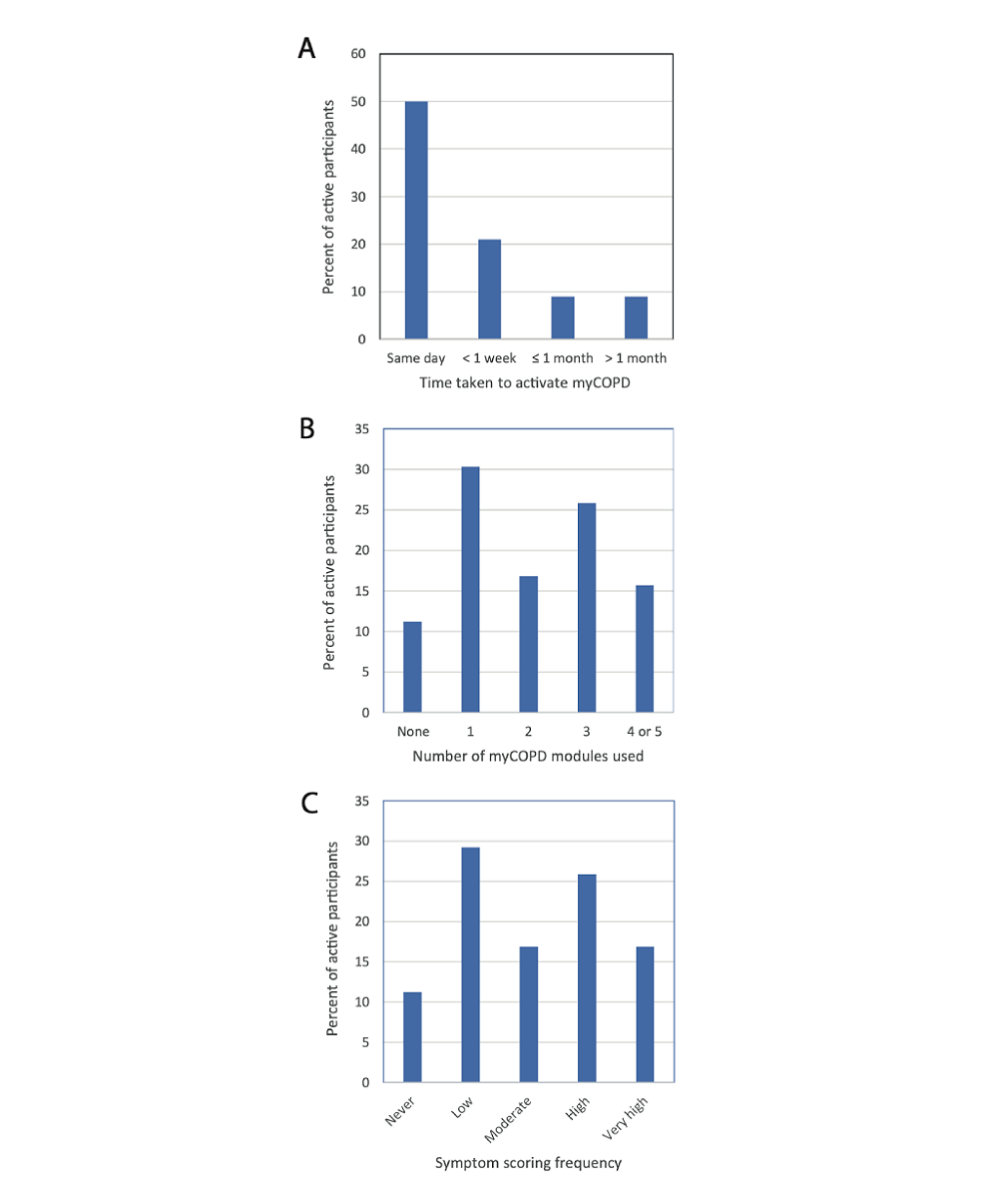
myCOPD engagement. Bar charts showing (A) time taken to activate myCOPD account following enrollment, (B) participant module usage, and (C) frequency of entering symptom scores. COPD: chronic obstructive pulmonary disease.

A total of 89 out of 113 (78.8%) participants activated myCOPD, with 56% (50/89) of them doing so on the day of enrollment and 90% (80/89) doing so within 1 month (Figure 2, A). Most active participants used at least one module and entered their symptom scores at least once (Figure 2, B and C; n=79, 89%). A total of 10 (11%) participants activated myCOPD but used no modules. Overall, 57% (n=51) of active participants recorded their CAT score one or more times, 39% (n=35) initiated pulmonary rehabilitation training, 24% (n=21) viewed educational course material, and 10% (n=9) watched at least one inhaler technique video. Out of 89 participants, 15 (17%) were very high users based on symptom scoring frequency (Figure 2, C), and 14 of these (93%) also used four or five myCOPD modules (Figure 2, B), suggesting a discrete subgroup of highly engaged users. There was no overall correlation between myCOPD engagement and participant age, SIMD status, or rurality (Multimedia Appendices 1 and 2).

**Figure 3:**
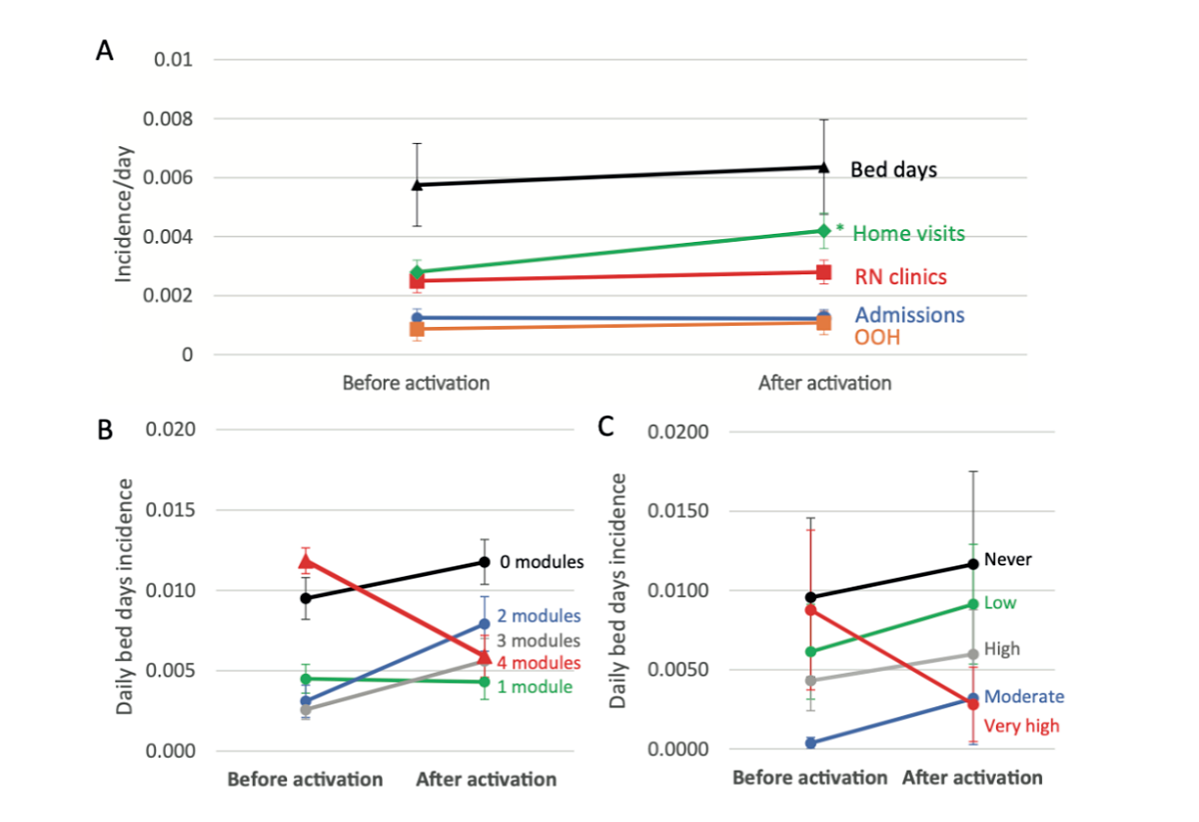
Daily incidence of health service usage before and after myCOPD activation. (A) Average daily incidence of health service use among all participants before and after myCOPD activation. Subgroup analysis of average daily inpatient bed day use before and after myCOPD activation according to (B) module usage and (C) frequency of symptom scoring. COPD: chronic obstructive pulmonary disease; OOH: out-of-hours; RN: registered nurse.

To evaluate myCOPD effectiveness, we quantified the daily incidence of inpatient bed days, hospital admissions, home visits, clinic appointments, and out-of-hours care provision for an average of 375 (SD 32) days before and 239 (SD 46) days after myCOPD activation, for a total of 69,211 participant days (47,972 days before and 21,239 days after). There were no significant differences in hospital admissions, inpatient bed days, or other health service use before or after myCOPD activation, apart from a modest increase in home visits consistent with previous telemonitoring studies [15] (Figure 3, A). Even after excluding participants who did not activate their license, there remained no significant difference for any of the categories.

Subgroup analysis results can be seen in Multimedia Appendix 3, A-H. Although underpowered, subgroup analysis based on either module usage (Figure 3, B; Multimedia Appendix 3, E) or symptom scoring frequency (Figure 3, C; Multimedia Appendix 3, F) did identify trends toward reduced inpatient bed days and hospital admissions for highly engaged users. There were also increased home visits in all subgroups after myCOPD activation regardless of module usage or symptom scoring frequency (Multimedia Appendix 3, A and B). No other trends in health service use were observed based on subgroup analysis regarding clinic appointments (Multimedia Appendix 3, C and D) and out-of-hours contacts (Multimedia Appendix 3, G and H).

## Discussion

This study is the longest and largest evaluation of the digital health self-management technology myCOPD to date, the only one involving a predominantly remote and rural population, and the first to recruit patients from within community care settings using a pragmatic approach. Enrollment and engagement with myCOPD was popular, with 78.8% (89/113) of participants activating the technology and 89% (79/89) of these participants using at least one module or entering their symptom scores at least once. Only 14.3% (20/140) of people approached declined to participate in the study, and there was no correlation between participant enrollment, activation, or engagement and either age, socioeconomics, rurality, or disease severity, suggesting that these are not significant barriers to using myCOPD. This finding may help mitigate perceived risks of increased health inequalities associated with the use of digital health technologies as part of routine care provision.

Despite high levels of activation, myCOPD did not reduce overall demands on health services. These findings are consistent with the limited evidence supporting the use of COPD self-management technologies, but they contrast with previous studies involving myCOPD [8-11]. There are several possible explanations for this difference. First, our study involved community-based recruitment of stable patients with COPD irrespective of exacerbation frequency, whereas other trials recruited hospital-based patients immediately following an acute exacerbation where motivation to engage in self-management may be greater. Second, previous studies collected data for only 90 days and, therefore, evaluated acute rather than long-term myCOPD benefits [9]. Finally, it remains possible that cultural or socioeconomic differences between rural and urban participants might influence myCOPD engagement and impact. Our results highlight the need for further evaluation of myCOPD and other digital health technologies ahead of their widespread procurement and adoption as part of routine health services. It may be that myCOPD can function as an effective tool in reducing COPD exacerbations when offered to participants in hospital and at a time of crisis, whereas it may not function in this manner when offered to patients in the community who are not actively in crisis or experiencing an exacerbation.

Despite no overall reduction in health service use, we did observe trends toward reduced hospital admissions and inpatient bed days in a subgroup of highly engaged users. This suggests the technology may be clinically beneficial if it is highly used and suggests that a greater emphasis is needed for understanding the motivation to use digital self-management tools and how to promote increased, meaningful user engagement. Paradoxically, previous studies indicate that those patients who may benefit most from engaging with digital self-management technologies are the least likely to do so [16]. Our observation that highly engaged myCOPD users were indistinguishable in terms of age, socioeconomics, rurality, or disease severity suggests that the factors driving meaningful user engagement are complex and require further attention. This will necessitate increased collaboration among a wide group of stakeholders, including patients, throughout all stages of digital health technology design, development, and testing.

One potential limitation of this study involves differences in the amount of data we collected before versus after myCOPD activation (47,972 participant days before and 21,239 after). Our original design involved collecting an equivalent quantity of data before and after myCOPD activation, but a decision was made to terminate the study on March 1, 2020, due to the emergent COVID-19 pandemic. We mitigated the impact of this change by evaluating data according to daily rather than annual individual health service usage. Interestingly, and despite cessation of formal data collection, we observed increased myCOPD engagement among many participants after March 2020, which may reflect changes in health behavior when access to face-to-face services was limited. A further impact of the COVID-19 pandemic was our limited ability to evaluate the effect of seasonality on exacerbation frequency, and it is possible that the inclusion of data beyond March 2020 may have resulted in different outcomes.

In conclusion, although our study does not support implementation of myCOPD to reduce health service demands on NHS Highland on a population-wide basis, our results do indicate that some highly engaged patients may derive benefits. Thus, individuals can be encouraged to individually adopt myCOPD as part of their self-management care should they find it useful. Further research is needed to understand what motivates some individuals to engage with digital health technologies, in order to facilitate the design and development of clinically and economically effective self-management tools.

## Appendix

Multimedia Appendix 1 Comparison of myCOPD engagement and patient demographics. Average daily symptom scoring frequency according to participant age (top), SIMD quintile (middle), and rurality score (bottom). Trendline and *R*^2^ values are representative of all participant data. COPD: chronic obstructive pulmonary disease; SIMD: Scottish Index of Multiple Deprivation.

Multimedia Appendix 2 Comparison of myCOPD engagement and patient demographics. myCOPD module usage according to participant age (top), SIMD quintile (middle), and rurality score (bottom). Trendline and *R*^2^ values are representative of all participant data. COPD: chronic obstructive pulmonary disease; SIMD: Scottish Index of Multiple Deprivation.

Multimedia Appendix 3 Subgroup analysis of health service usage relative to myCOPD engagement. Average daily home visits (A, B), registered nurse (RN) clinics (C, D), hospital admissions (E, F), and out-of-hours (OOH) care (G, H) according to myCOPD module usage (A, C, E, G) or symptom scoring frequency (B, D, F, H) for all participant subgroups. COPD: chronic obstructive pulmonary disease.

## Abbreviations

CAT: COPD Assessment Test
COPD: chronic obstructive pulmonary disease
NHS: National Health Service
SIMD: Scottish Index of Multiple Deprivation

## References

[1] (2021). Lung disease in the UK - Big picture statistics. British Lung Foundation.

[2] Chronic obstructive pulmonary disease (COPD) statistics. British Lung Foundation.

[3] Trueman D, Woodcock F, Hancock E (2017). Estimating the Economic Burden of Respiratory Illness in the UK.

[4] Raju S, Keet CA, Paulin LM, Matsui EC, Peng RD, Hansel NN, McCormack MC (2019). Rural residence and poverty are independent risk factors for chronic obstructive pulmonary disease in the United States. Am J Respir Crit Care Med.

[5] Lenferink A, Brusse-Keizer M, van der Valk PD, Frith PA, Zwerink M, Monninkhof EM, van der Palen J, Effing TW (2017). Self-management interventions including action plans for exacerbations versus usual care in patients with chronic obstructive pulmonary disease. Cochrane Database Syst Rev.

[6] Murphy LA, Harrington P, Taylor SJ, Teljeur C, Smith SM, Pinnock H, Ryan M (2017). Clinical-effectiveness of self-management interventions in chronic obstructive pulmonary disease: An overview of reviews. Chron Respir Dis.

[7] myCOPD: The COPD app for controlling your symptoms. my mhealth.

[8] Bourne S, DeVos R, North M, Chauhan A, Green B, Brown T, Cornelius V, Wilkinson T (2017). Online versus face-to-face pulmonary rehabilitation for patients with chronic obstructive pulmonary disease: Randomised controlled trial. BMJ Open.

[9] North M, Bourne S, Green B, Chauhan AJ, Brown T, Winter J, Jones T, Neville D, Blythin A, Watson A, Johnson M, Culliford D, Elkes J, Cornelius V, Wilkinson TMA (2020). A randomised controlled feasibility trial of E-health application supported care vs usual care after exacerbation of COPD: The RESCUE trial. NPJ Digit Med.

[10] Crooks MG, Elkes J, Storrar W, Roy K, North M, Blythin A, Watson A, Cornelius V, Wilkinson TMA (2020). Evidence generation for the clinical impact of myCOPD in patients with mild, moderate and newly diagnosed COPD: A randomised controlled trial. ERJ Open Res.

[11] Shaw G, Whelan ME, Armitage LC, Roberts N, Farmer AJ (2020). Are COPD self-management mobile applications effective? A systematic review and meta-analysis. NPJ Prim Care Respir Med.

[12] (2018). Scottish Government Urban Rural Classification 2016.

[13] (2020). Scottish Index of Multiple Deprivation.

[14] (2017). Pocket Guide to COPD Diagnosis, Management, and Prevention: A Guide for Health Care Professionals. 2017 Report.

[15] Pinnock H, Hanley J, McCloughan L, Todd A, Krishan A, Lewis S, Stoddart A, van DPM, MacNee W, Sheikh A, Pagliari C, McKinstry B (2013). Effectiveness of telemonitoring integrated into existing clinical services on hospital admission for exacerbation of chronic obstructive pulmonary disease: Researcher blind, multicentre, randomised controlled trial. BMJ.

[16] Robbins R, Krebs P, Jagannathan R, Jean-Louis G, Duncan DT (2017). Health app use among US mobile phone users: Analysis of trends by chronic disease status. JMIR Mhealth Uhealth.

