# Supplementary figures and images for: Evaluation of myCOPD Digital Self-management Technology in a Remote and Rural Population: Real-world Feasibility Study
Source: JMIR Mhealth Uhealth. 2022 Feb 7;10(2):e30782. doi: 10.2196/30782 (PMC8861861; doi:10.2196/30782)

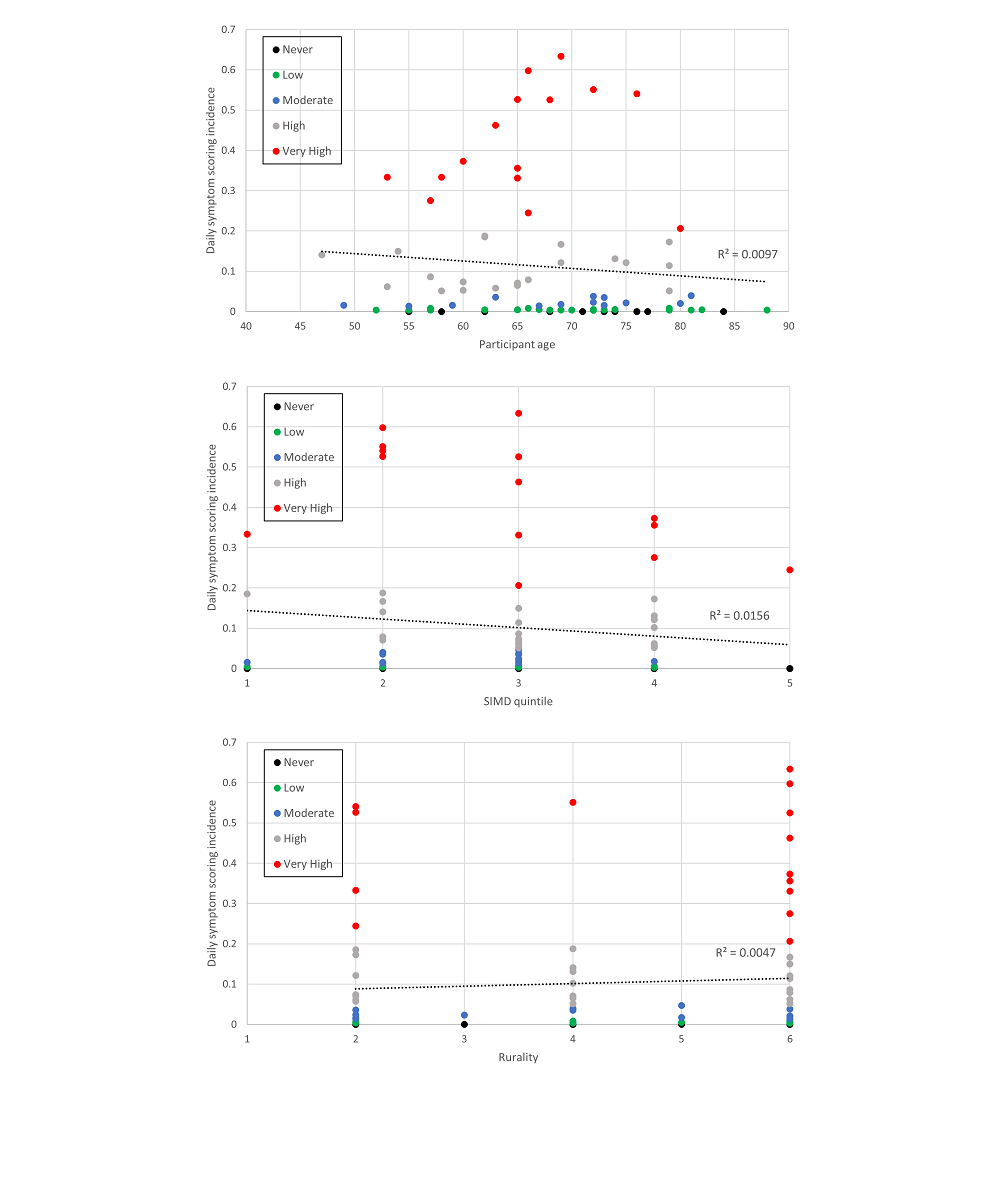

Supplement: Multimedia Appendix 1 [file mhealth_v10i2e30782_app1.png]

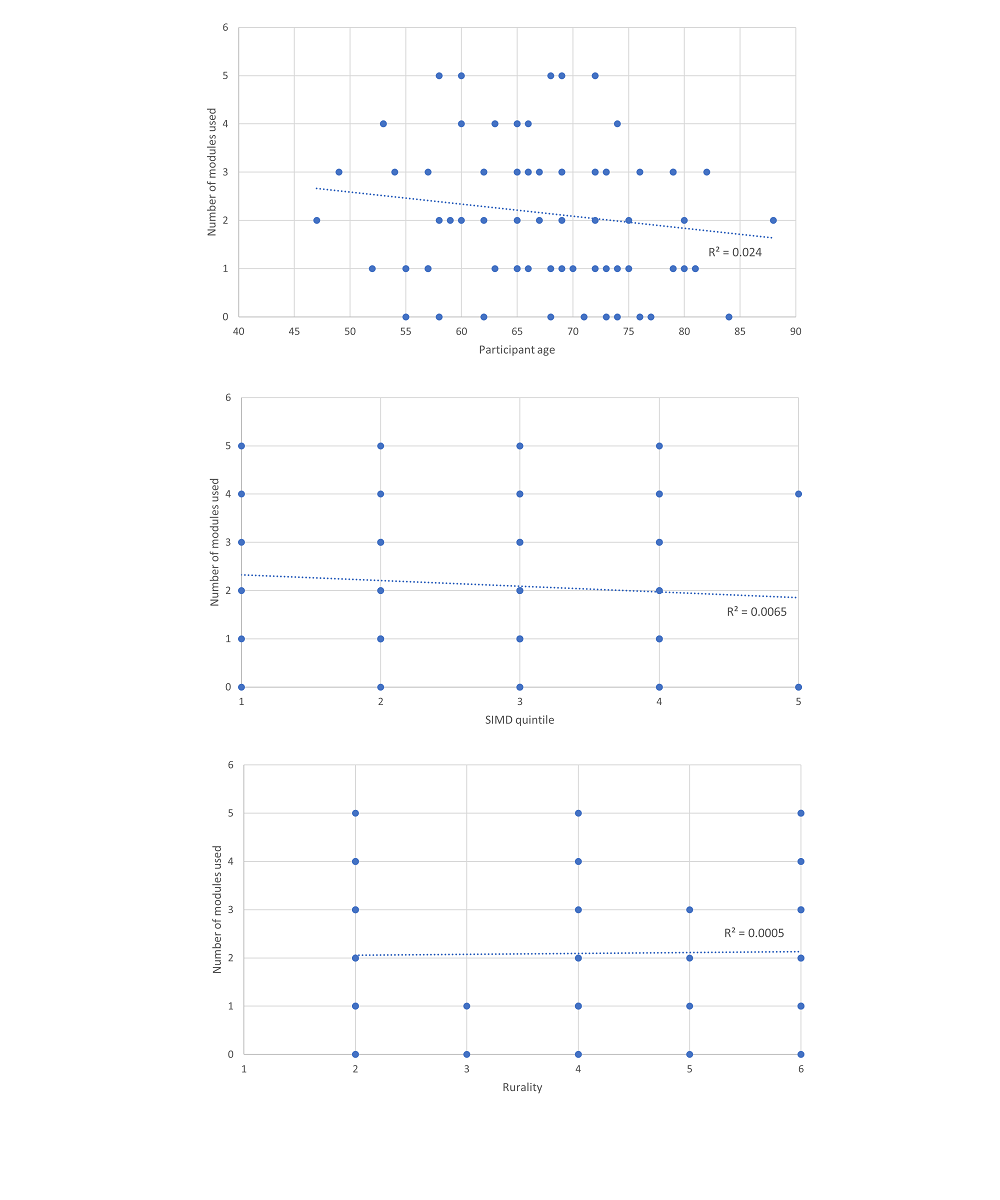

Supplement: Multimedia Appendix 2 [file mhealth_v10i2e30782_app2.png]

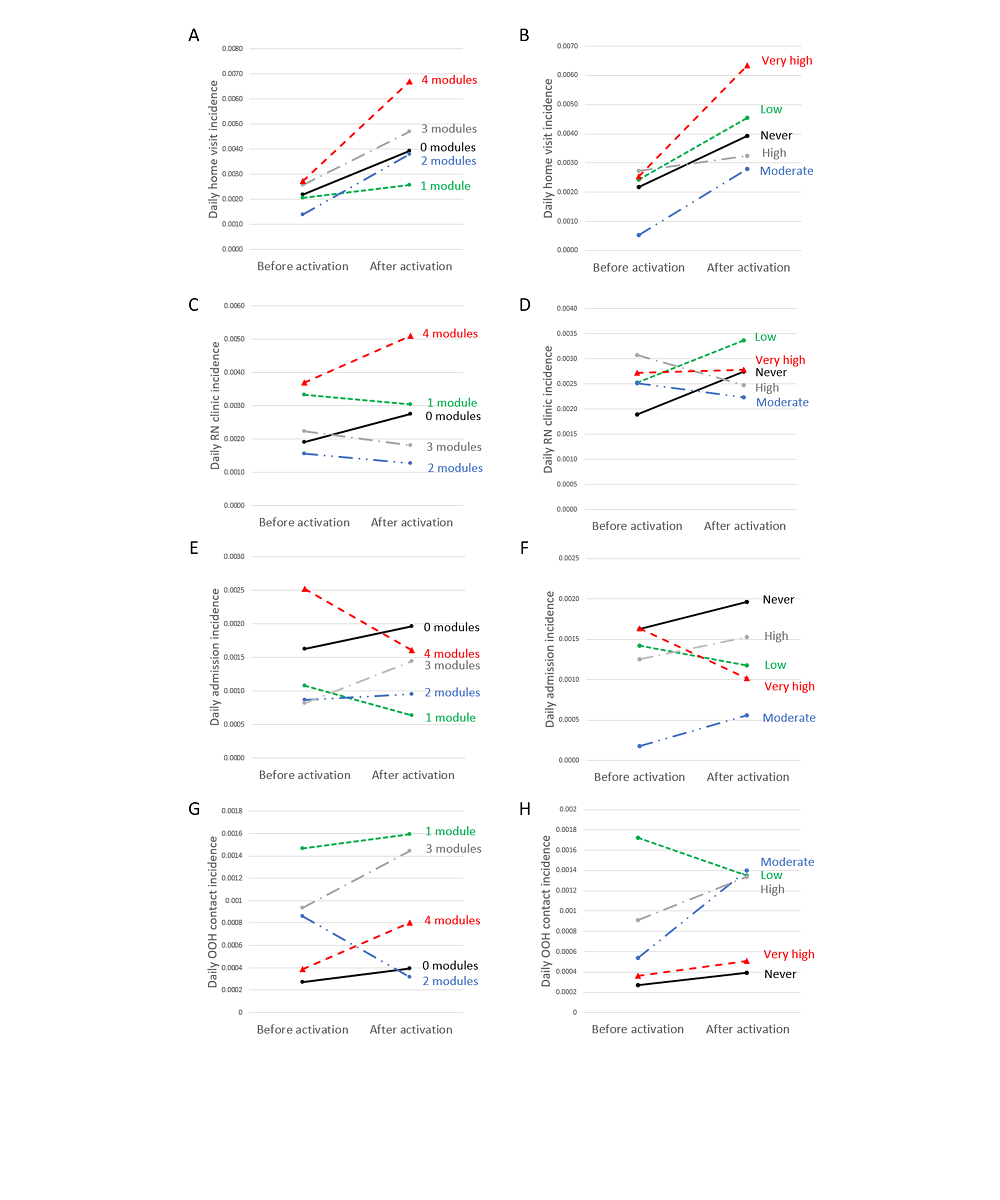

Supplement: Multimedia Appendix 3 [file mhealth_v10i2e30782_app3.png]
